# Biochemical screening for SARS-CoV-2 main protease inhibitors

**DOI:** 10.1371/journal.pone.0240079

**Published:** 2020-10-06

**Authors:** Camila Coelho, Gloria Gallo, Claudia B. Campos, Leon Hardy, Martin Würtele

**Affiliations:** 1 Department of Science and Technology, Federal University of São Paulo, São José dos Campos, Brazil; 2 Department of Physics, University of South Florida, Tampa, FL, United States of America; Stanford University, UNITED STATES

## Abstract

The Severe Acute Respiratory Syndrome Corona Virus 2 (SARS-CoV-2) pandemic represents a global challenge. SARS-CoV-2's ability to replicate in host cells relies on the action of its non-structural proteins, like its main protease (M^pro^). This cysteine protease acts by processing the viruses' precursor polyproteins. As proteases, together with polymerases, are main targets of antiviral drug design, we here have performed biochemical high throughput screening (HTS) with recombinantly expressed SARS-CoV-2 M^pro^. A fluorescent assay was used to identify inhibitors in a compound library containing known drugs, bioactive molecules and natural products. These screens led to the identification of 13 inhibitors with IC_50_ values ranging from 0.2 μM to 23 μM. The screens confirmed several known SARS-CoV M^pro^ inhibitors as inhibitors of SARS-CoV-2 M^pro^, such as the organo-mercuric compounds thimerosal and phenylmercuric acetate. Benzophenone derivatives could also be identified among the most potent screening hits. Additionally, Evans blue, a sulfonic acid-containing dye, could be identified as an M^pro^ inhibitor. The obtained compounds could be of interest as lead compounds for the development of future SARS-CoV-2 drugs.

## Introduction

The agent behind the Coronavirus Disease 2019 (COVID-19) pandemic, SARS-CoV-2, is an RNA virus from the betacoronavirus genus [1, 2]. The genome of this virus has about 88% identity to coronaviruses from bats, but only 79% to SARS-CoV and 50% to MERS-CoV viruses [3]. SARS-CoV-2 shares the typical gene array of coronaviruses. About two thirds of the genome is occupied by orf1ab that encodes the non-structural proteins, while the remaining region next to the 3’ end encodes the structural proteins [3]. Orf1ab is translated into two polyproteins. They are processed by the virus’s main protease M^pro^ (also termed 3CL^pro^ because of its homology to the picornavirus 3C protease) and a second papain-like protease (PL^pro^) [4]. The structure of M^pro^ from SARS-CoV-2, a protein with 96% sequence identity to M^pro^ from SARS-CoV, was recently solved [5, 6]. It consists of a dimeric 6-stranded β-barrel chymotrypsin-like fold with homology to the monomeric picornavirus 3C protease fold. The enzyme’s active site contains a cysteine-histidine catalytic dyad. M^pro^ has an additional C-terminal helical domain and an N-terminal chain of amino acids termed the “N-finger”. The helical domain, together with the N-finger amino acids, form a dimerization interaction surface for a second M^pro^ protomer. The resulting dimer has an estimated dissociation constant of approximately 2.5 μM [6]. The N-finger chain is important for activity as it stabilizes part of the adjacent monomer’s S1 binding pocket. M^pro^ is thought to specifically cleave the viral polyprotein 1ab at 11 cleavage sites. The sequence recognized contains in most cases Leu-Gln-(Ser/Ala/Gly) with cleavage occurring after the Gln residue [5–7]. Although currently several promising therapeutic strategies against SARS-CoV-2 are in development [8], no established COVID-19 drug or vaccine exists. By the end of May 2020 worldwide statistics accounted for more than 5.8 million confirmed infections and 360 thousand deaths due to the effects of COVID-19 (https://coronavirus.jhu.edu/map.html). As viral proteases, following polymerases, are the most prominent targets for antiviral drug design [9], here we describe initial biochemical screenings with recombinant purified SARS-CoV-2 M^pro^ performed in order to define possible candidates which could serve as lead compounds for the design of future COVID-19 therapies.

## Results and discussion

In order to contribute to the ongoing worldwide research and development efforts to contain COVID-19, we cloned, expressed recombinantly in *E*.*coli* BL21(DE3) and purified an important drug target of SARS-CoV-2, its main protease (M^pro^). After His-tag cleavage, screens were carried out in concentrations of 1 μM M^pro^ and 10 μM of a previously described fluorogenic substrate-peptide MCA-AVLQSGFR-K(Dnp)-K-NH2 [5]. Screens of a library containing 2400 drugs and drug-related molecules as well as natural products led to several interesting hits.

**Fig 1:**
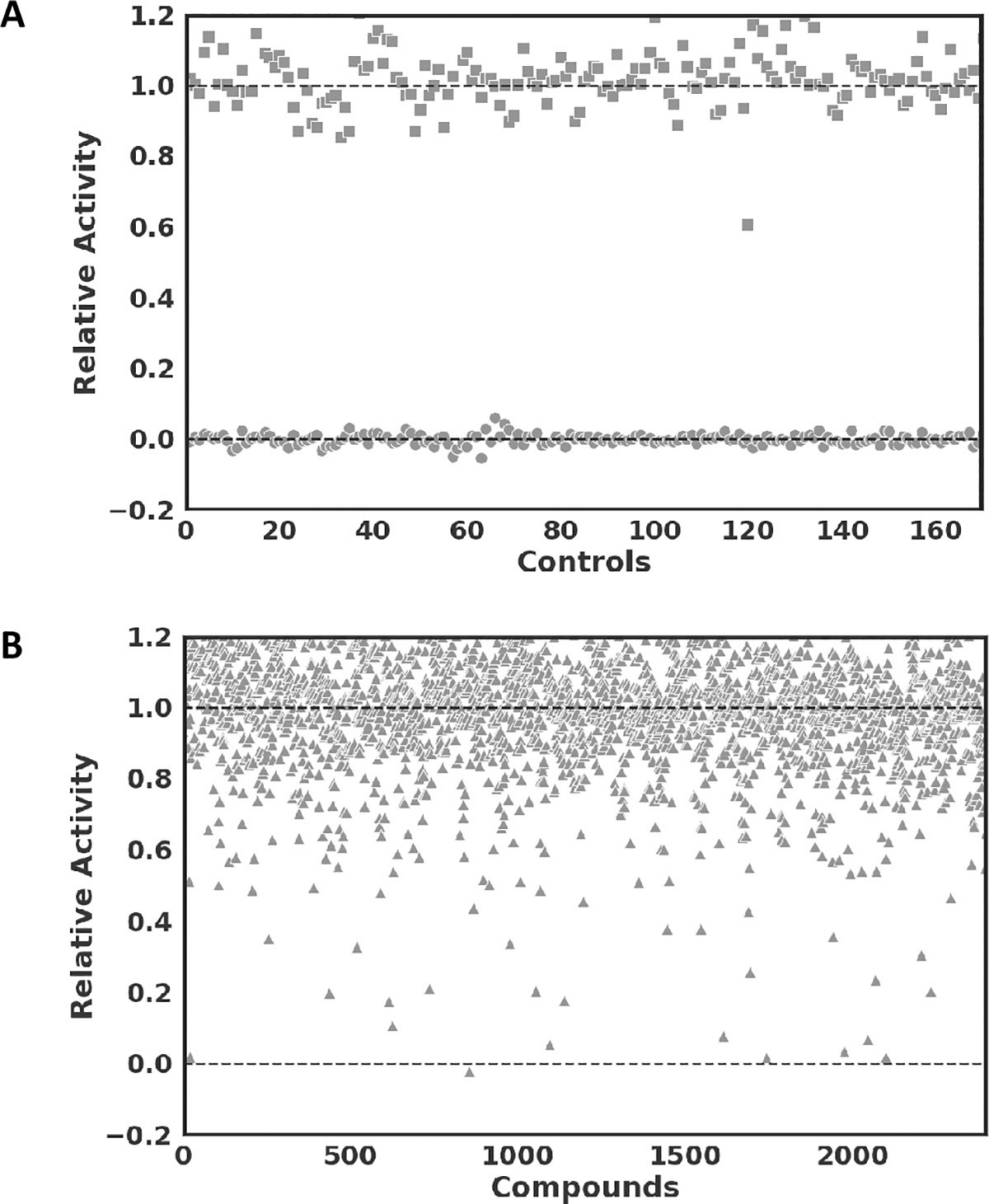
High throughput screen. (A) Relative activities, defined as initial reaction rates of assays normalized by average initial reaction rates of negative controls (squares, fluorogenic peptide substrate assays with 10 μM substrate and 1μM recombinant M^pro^ without inhibitors) and positive controls (circles, assays including 40 μM tannic acid as control). (B) High throughput screen results with relative activities of all tested compounds (triangles).

As control experiments to validate the screenings, enzyme substrate assays without inhibitors (negative control) as well as enzyme substrate assays with tannic acid, a known inhibitor of SARS-CoV M^pro^ (positive control) [10], were used. The relative activity of the assay was defined as the quotient between the initial reaction rates of the experiments and the negative controls. As a result, an average relative activity of 1.0 (Standard deviation, SD = 0.08) for the negative and 0.0 (SD = 0.014) for the positive controls was obtained. Control experiments thus showed a significant separation of relative activity of the negative and positive controls (**Fig 1A**) leading to an acceptable HTS Z’ value [11] of 0.72. The average value of the relative activities of the compound screening assays was 0.98 (SD = 0.2, **Fig 1B**). After the screenings, 13 of the most prominent hits were selected for confirmation and further biochemical characterization based on a cut-off relative activity below 0.2. These compounds, together with their corresponding half-maximum inhibitory concentration (IC_50_) values are shown in **Table 1**.

**Table 1:** IC_50_ and K_i_ values of SARS-Cov-2 M^pro^ inhibitors.

Compound	IC_50_ (μM)*	K_i_ (μM)*
Thimerosal (**1**)	0.6 ± 0.1	0.6 ± 0.2
Phenylmercuric acetate (**2**)	0.4 ± 0.06	0.11 ± 0.03
Bronopol (**3**)	4.4 ± 0.6	2.5 ± 0.3
Tannic acid (**4**)	2.1 ± 0.2	1.4 ± 0.14
Hematoporphyrin (**5**)	3.9 ± 0.6	5.9 ± 0.5
3,4-Didesmethyl-5-deshydroxy-3'-ethoxyscleroin (**6**)	10.6 ± 1.3	5.6 ± 0.5
2,3,4-Trihydroxy-4'-ethoxybenzophenone (**7**)	9.0 ± 1.5	ND
Chloranil (**8**)	4.1 ± 0.8	ND
Plumbagin (**9**)	17.1 ± 9	ND
Vanitiolide (**10**)	4.6 ± 0.6	ND
Evans blue (**11**)	0.2 ± 0.06	0.21 ± 0.02
Chicago Sky Blue (**12**)	7.7 ± 1.6	1.3 ± 0.2
Protoporphyrin IX	23 ± 2.4	ND

*Error values are expressed as standard error of the mean (SEM).

ND-not determined.

For M^pro^ from SARS-CoV and SARS-CoV-2, several interesting inhibitors have been reported. Inhibitors of SARS-CoV M^pro^ discovered by high throughput screening include diverse compounds characterized with K_i_ values ranging from 0.5 μM to 75 μM [10, 12–16]. From these obtained compounds, esculetin-4-carboxylic acid ethyl ester (IC_50_ = 46 μM in M^pro^ inhibition assays), a coumarin derivative and natural product, demonstrated an EC_50_ of 112 μM (median toxic concentration TC_50_>800μM) in Vero-cell SARS-CoV assays [13] and MP576 (IC_50_ = 2.5 μM), a quinolinecarboxylate, demonstrated an EC_50_ of 7 μM (TC_50_>50μM) [15, 17], thus validating the M^pro^ biochemical screening approach for the development of SARS-CoV drugs. Additionally, several other notable SARS-CoV M^pro^ inhibitors, like TG-0205221 a peptidomimetic covalent inhibitor with a K_i_ value of 53 nM [18] (EC_50_ = 0.6 μM, TC_50_>20 μM in cellular assays) and boronic acid derivatives with K_i_ values up to 40 nM [19], among several others, have been published. Regarding SARS-CoV-2 M^pro^ inhibition, several promising lead compounds have been reported, like e.g. ebselen (IC_50_ = 0.67 μM, EC_50_ = 4.67 μM, LD_50_> 4,600 mg/kg in rats), tideglusib (IC_50_ = 1.55 μM) Carmofur (IC_50_ of 1.82 μM) [5]; a peptidomimetic α-ketoamide with an IC_50_ value of 0.67 μM for SARS-CoV-2 M^pro^ and an EC_50_ value of 4 to 5 μM in human cell culture experiments that covalently binds to the catalytic cysteine as shown in X-ray diffraction experiments [6]; two peptidomimetic compounds with an aldehyde reactive group that covalently binds the catalytic cysteine with an IC_50_ of 40 nM and 53 nM (EC_50_ values of 0.53 μM and 0.72 μM) [20] and atazanavir (EC_50_ = 2.0 μM) [21].

**Fig 2:**
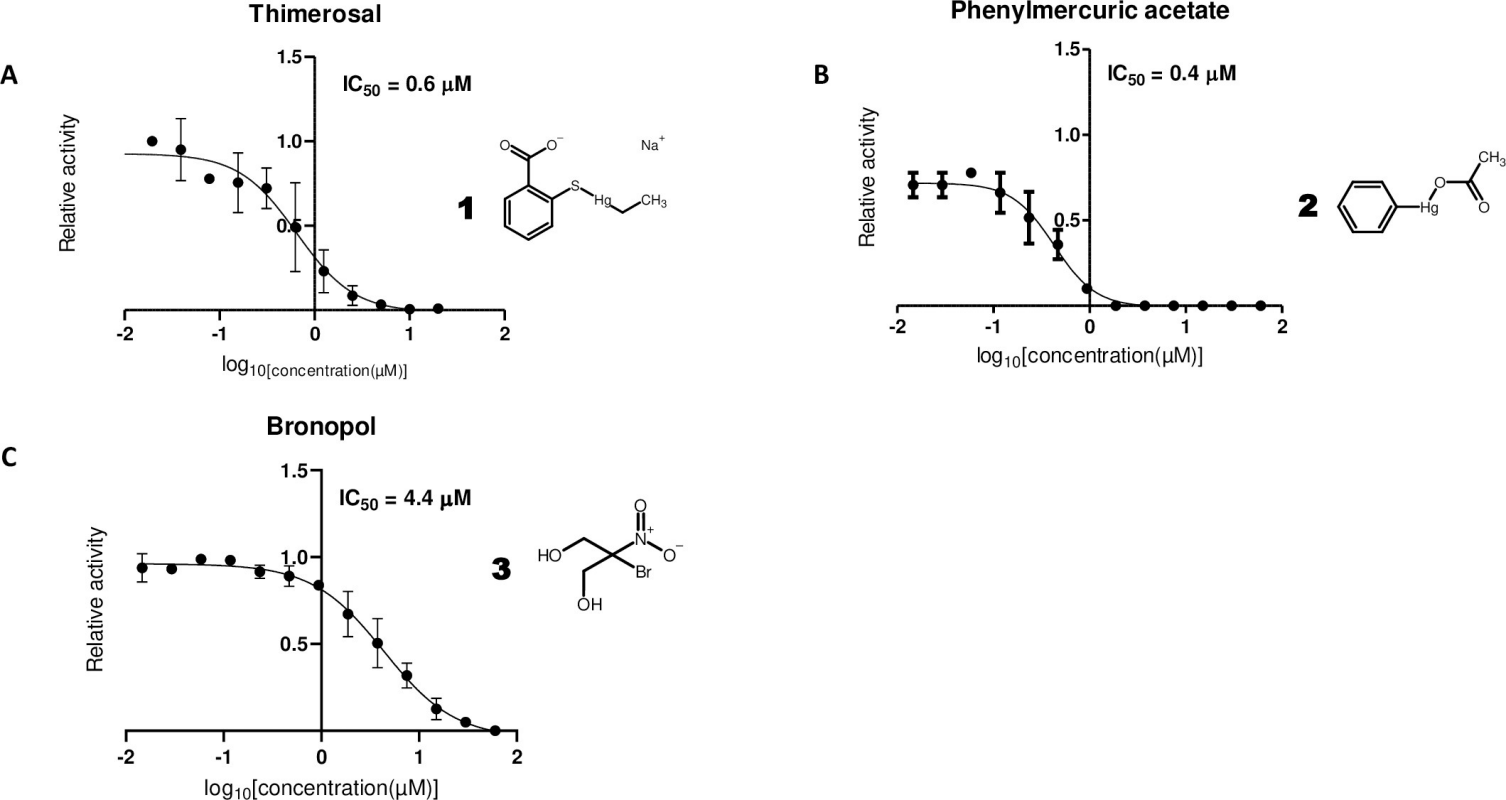
Effective compounds with known action against cysteines. Dose-response curves for (A) thimerosal, (B) phenylmercuric acetate, (C) bronopol. Half-maximum inhibitory concentration (IC_50_) values were determined by nonlinear regression using 10 μM substrate and 0.5 μM enzyme with varying concentrations of inhibitors.

In this work, it was possible to confirm thimerosal (**1**, IC_50_ = 0.6 μM, **Fig 2A**) and phenylmercuric acetate (**2**, IC_50_ = 0.4 μM, **Fig 2B**), both previously described as SARS-CoV M^pro^ inhibitors [14], as SARS-CoV-2 M^pro^ inhibitors. This common mode of inhibition can be expected, as SARS-CoV-2 and SARS-CoV M^pro^ share an overall amino acid identity of 96%, with practically all amino acids from the active site being conserved. Thimerosal is an organometallic compound originally used as an antiseptic (e.g. Merthiolate) and preservative in vaccines, pharmaceutical products as well as cosmetics [22]. Phenylmercuric acetate is another organo-mercuric compound, used as preservative in paints and as a disinfectant [23]. Thimerosal was initially identified together with phenylmercuric acetate in a HTS as a SARS-CoV M^pro^ inhibitor. This result led to the further identification of four other Hg-containing compound as well as several presumably less toxic Zn rather than Hg-containing compounds, with K_i_ values ranging from 0.17 μM to 1.4 μM [14]. Both thimerosal and phenylmercuric acetate and other Hg-containing molecules are thought to have antibacterial properties by their capacity to bind thiol groups in proteins [23], like the catalytic cysteine of M^pro^. With regards to other viral infections, very low doses of thimerosal have been additionally found to modulate and promote the host's immune response, promoting Th2-cell responses and inhibiting proinflammatory cytokines and chemokines [24], which could provide further benefits in the treatment of COVID-19.

Another compound identified as SARS-CoV-2 M^pro^ inhibitor in this work is bronopol (**3**, 2-bromo-2-nitropropane- 1,3-diol, **Fig 2C**, IC_50_ = 4.4 μM). Bronopol is a wide range antibacterial agent used as a preservative in e.g. cosmetics and pharmaceutical products [25], which is thought to deactivate enzymes by its oxidative effect on thiol groups [26]. The identification of metal-conjugate inhibitors and thiol oxidizing compounds indicates that approaches that take advantage of the fact that M^pro^ is a cysteine protease are an interesting option to be exploited.

**Fig 3:**
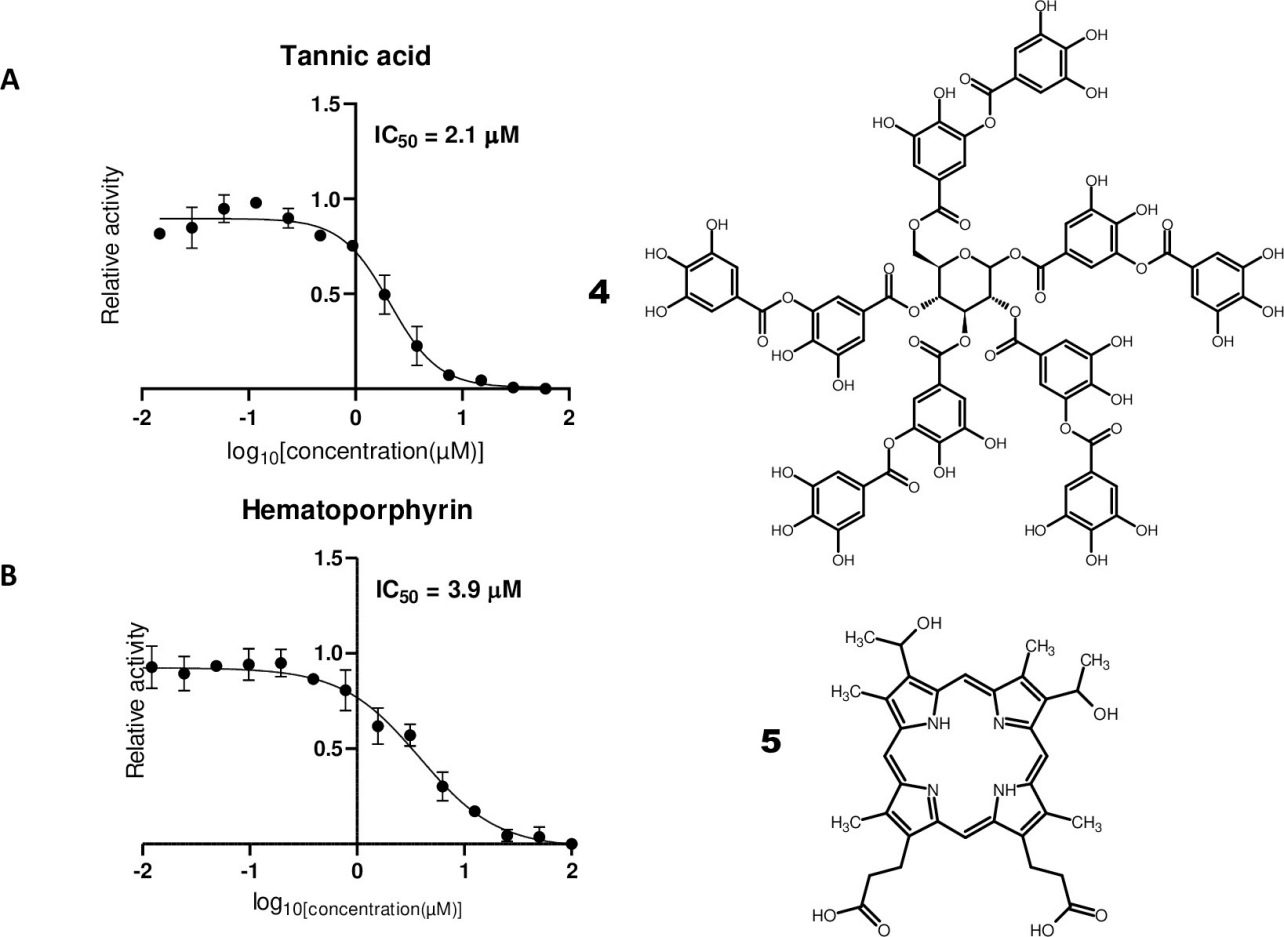
Natural products with inhibitory activity on SARS-CoV-2 M^pro^. Dose-response curves for (A) tannic acid and (B) hematoporphyrin. IC_50_ values were determined as stated in **Fig 1**.

We could additionally confirm tannic acid (**4**, **Fig 3A**), which has an IC_50_ of 3 μM for SARS-CoV M^pro^ [10], as a SARS-CoV-2 M^pro^ inhibitor with an IC_50_ of 2.1 μM. Tannic acid, a hydrolysable tannin, is a polyphenolic compound formed by a glucose moiety and gallic acid. Several enzymes have been shown to be inhibited by tannic acid, including proteases [27]. Due to these properties, tannic acid was used successfully in this work as a positive control.

Surprisingly hematoporphyrin (**5**, **Fig 3B**, IC_50_ = 3.9 μM) was a hit in the SARS-CoV-2 M^pro^ screens. Hematoporphyrin is a derivative of hemoglobin’s protoporphyrin IX ring system. Consequently, protoporphyrin IX was additionally tested with the M^pro^ assay and an IC_50_ value of 23 μM was obtained. Hematoporphyrin is used in photodynamic therapy [28] and was formerly used as an antidepressant [29]. Although this finding could potentially indicate that M^pro^ mediates a hypothetical link between COVID-19 and hematological disorders, like virus induced porphyria [30] and SARS-Cov-2 induced coagulation disorders [31, 32] the evidence presented here is far too preliminary and speculative. Furthermore, both substances have been described as being so called promiscuous compounds in HTS [33]. In this sense, hematoporphyrin could catalyze as a photosensitizer through free radical generation inactivation by oxidation of the catalytic cysteine of M^pro^ [34, 35]. Thus, further work has to be carried out to corroborate whether hematoporphyrin is indeed a specific inhibitor of SARS-Cov-2 M^pro^ and eventually a mediator of hematological disorders.

**Fig 4:**
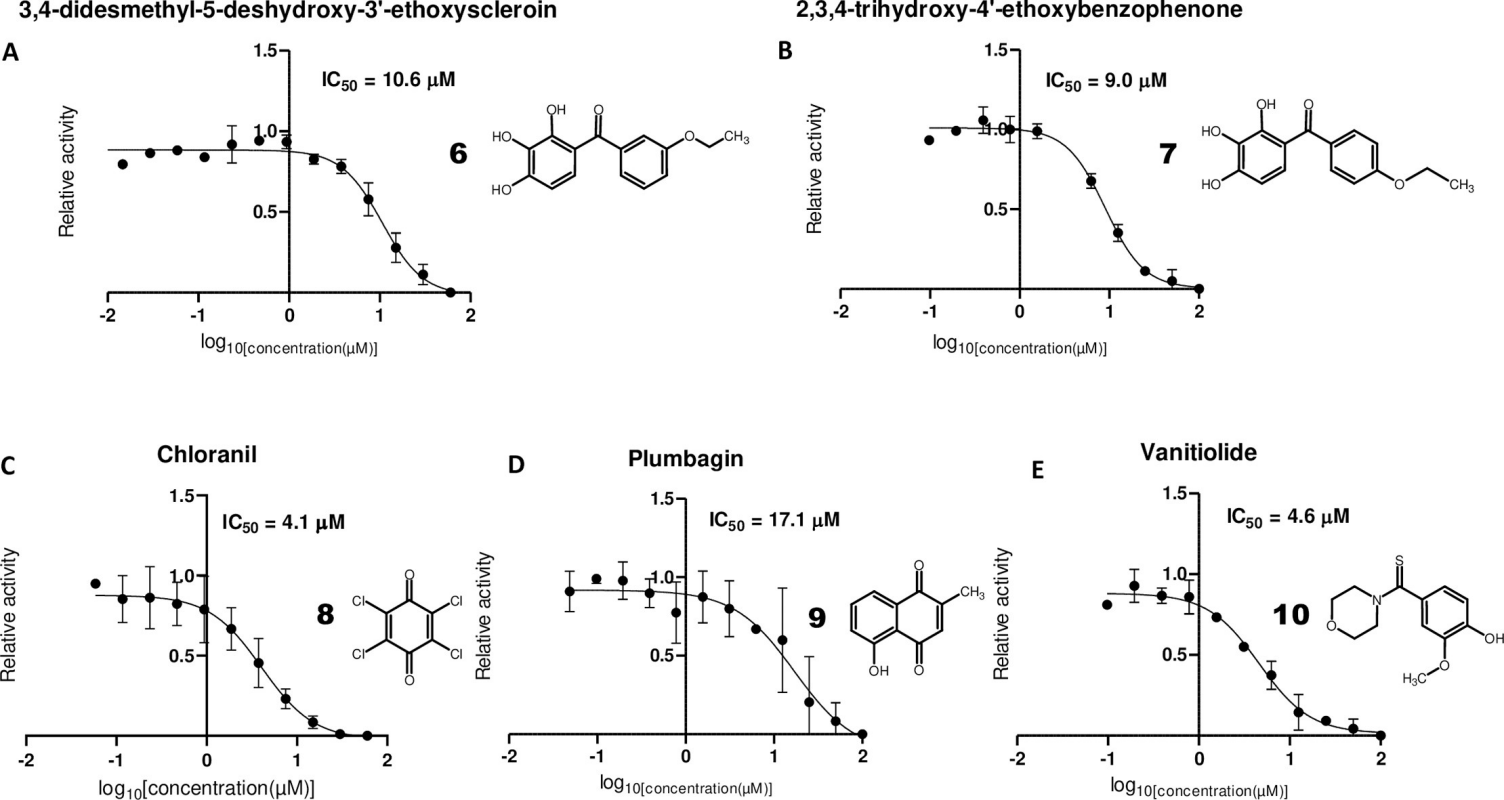
Further effective inhibitors of SARS-CoV-2 M^pro^. Dose-response curves for (A) 3,4-didesmethyl-5-deshydroxy-3’-ethoxyscleroin, (B) 2,3,4-trihydroxy-4’-ethoxybenzophenone, (C) chloranil, (D) plumbagin and (E) vanitiolide. IC_50_ values were determined as stated in **Fig 1**.

Two other interesting related M^pro^ inhibitors obtained were 3,4-didesmethyl-5-deshydroxy-3'-ethoxyscleroin (**6**, IUPAC-Name: (3-ethoxyphenyl)-(2,3,4-trihydroxyphenyl)methanone, **Fig 4A**) and its isomer 2,3,4-trihydroxy-4'-ethoxybenzophenone (**7**, IUPAC-Name: (4-ethoxyphenyl)-(2,3,4-trihydroxyphenyl)methanone, **Fig 4B**). Whereas the 3-ethoxyphenyl isomer (**6**) had an IC_50_ of 10.6 μM, the 4-ethoxyphenyl (**7**) isomer had a similar IC_50_ of 9 μM. Interestingly, other benzophenone derivatives have been reported as inhibitors of protozoan cysteine proteases [36, 37]. The behavior of benzophenones as free radical generators upon UV light stimulation could be a possible explanation for their cysteine protease inhibitory activity [35]. However, the specificity of this effect has to be further elucidated, as other benzophenones present in the library, like 2,3,4'-trihydroxy-4-methoxybenzophenone showed no significant inhibitory activity.

**Fig 5:**
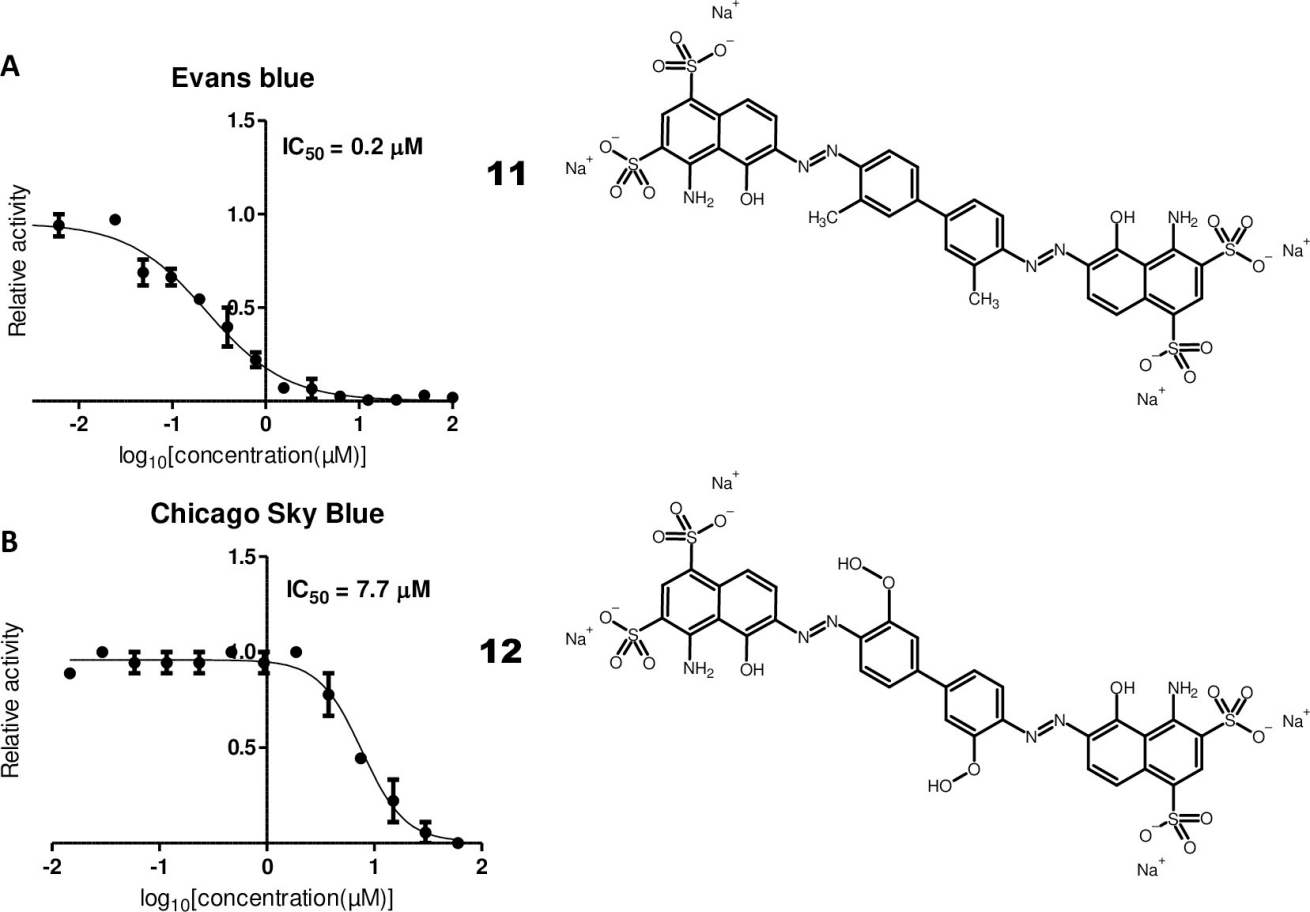
Sulfonic acid-containing dyes with inhibitory activity on SARS-CoV-2 M^pro^. Dose-response curves for (A) Evans blue and (B) Chicago Sky Blue. IC_50_ values were determined as stated in **Fig 1**.

Two quinones, chloranil (**8**, **Fig 4C**, IC_50_ = 4.1 μM) and plumbagin (**9**, **Fig 4D**, IC_50_ = 17.1 μM) were also identified in the screens. As quinones are mild oxidizing agents, their activity could be related to oxidation of the catalytic cysteine of M^pro^ [38]. Another compound, vanitiolide (**10**, **Fig 4E**, IUPAC-Name: 4-hydroxy-3-methoxyphenyl)(4-morpholinyl)methanethione, IC_50_ = 4.6 μM), a choleretic drug, was also identified in the SARS-CoV-2 M^pro^ screens. Finally, two related sulfonic acid-containing dyes, Evans blue (**11**, **Fig 5A**, IC_50_ = 0.2 μM) and Chicago Sky Blue (**12**, **Fig 5B**, IC_50_ = 7.7 μM) could be identified in the screens. Interestingly, both compounds have been previously reported to inhibit human immunodeficiency virus (HIV) in cellular assays as well HIV’s reverse transcriptase in biochemical assays [39] and Evans blue has been reported as a hepatitis B virus (HBV) inhibitor in cellular assays [40].

For the top inhibiting compounds, thimerosal (**1**), phenylmercuric acetate (**2**), bronopol (**3**), tannic acid (**4**), hematoporphyrin (**5**), 3,4-didesmethyl-5-deshydroxy-3'-ethoxyscleroin (**6**), Evans blue (**11**) and Chicago Sky Blue (**12**) detailed kinetical experiments were carried out (**S1 Fig in S1 File**). The resulting K_i_ values are shown in **S1 Table in S1 File**. The values are, as expected, mostly close to the obtained IC_50_ values. Evans blue (**11**), Chicago Sky Blue (**12**), phenylmercuric acetate (**2**) and tannic acid (**4**) show competitive inhibition, as indicated by the Dynafit analysis (SSQ, summed squared deviation between experimental data and theoretical model, and the related ΔAIC/ΔBIC, second order Akeike information criterion/ Bayesian information criterion [41]; **S1 Table in S1 File**). However, thimerosal (**1**), bronopol (**3**), hematoporphyrin (**5**), 3,4-didesmethyl-5-deshydroxy-3'-ethoxyscleroin (**6**) showed mixed or non-competitive inhibition.

As a whole, the obtained compounds here described are in an IC_50_ range from 0.2 to 23 μM (**Table 1**) that would justify further biochemical testing as well as testing in cellular assays which would confirm them as lead compounds for COVID-19 drug development. Although some are natural products and some have a record as pharmaceutical agents, which may accelerate their development, some have toxicity issues, which have to be carefully evaluated (**S2 Table in S1 File**). Emergence of COVID-19, with its huge human, social and economic costs and implications has certainly demonstrated the necessity for the development of novel antiviral drugs.

## Material and methods

### Expression and purification

The gene of SARS-CoV-2 M^pro^ (GenBank entry MT358641.1) was synthesized (GenScript, USA) and cloned into the pET21a expression plasmid. The resulting expression construct contains an N-terminal His-tag followed by a tobacco etch virus (TEV) protease cleavage site, so that the resulting protein after His-tag cleavage is the full-length native SARS-Cov-2 Mpro including two additional (Gly-Ser) N-terminal residues. The protein was expressed in *E*. *coli* BL21 (DE3) grown in Luria Bertani broth containing 50 μg/ml ampicillin at 37°C after induction with 0.5 mM isopropyl-ß-D-1-thiogalactopyranoside (IPTG) for 8 hours at 30°C. After harvesting by centrifugation, cells were disrupted with lysis buffer containing 50 mM Tris-HCl pH 8, 1% Brij 98, 300 mM NaCl, 5 mM imidazole, DNAse and lysozyme. The protein was purified from the soluble fraction using an ÄKTAprime Plus liquid-chromatography system (GE Healthcare) by affinity chromatography employing a 5 ml HisTrap Sepharose column (GE healthcare) using a 50 mM Tris-HCl pH 7.3, 150 mM NaCl buffer and a 5 mM to 500 mM imidazole gradient for elution. A second purification step was performed using size exclusion chromatography with a HiLoad 26/600 Superdex 75 prep-grade column (GE Healthcare) using a 50 mM Tris-HCl pH 7.3, 150mM NaCl buffer. Finally, the TEV protease [42] was used to cleave the His-Tag of the protein in 50 mM Tris-HCl pH 8, 1 mM DTT, 0.5 mM EDTA for 4 hours at 8°C. The protein was then purified to remove the His-tagged TEV protease and the cleaved affinity-Tag by a further step of Ni-affinity chromatography in 50 mM Tris-HCl pH 7.3, 150 mM NaCl buffer.

### Biochemical screening

The Spectrum Collection (Microsource Discovery Systems Inc.) compound library was screened using a Freedom EVO 150 liquid handler (Tecan Group Ltd.). Assays were performed in 50 mM Tris-HCl pH 7.3, 20% glycerol, 1mM EDTA pH 7.3 and 0.01% triton-X using 1 μM M^pro^, 40 μM compounds and 10 μM substrate-peptide (MCA-AVLQSGFR-K(Dnp)-K-NH2, Biomatik Corporation, Cambridge, Canada) [5] at 30°C after a compound incubation period of 10 minutes. The reaction was monitored using an excitation wavelength of 330 nm and an emission wavelength of 400 nm on an Infinite M200 plate reader (Tecan Group Ltd.).

### Biochemical characterization

IC_50_ values were determined using concentrations from 122 nM to 100 μM compounds and 0.5 μM M^pro^ with 10 μM substrate. All tests were carried out in triplicate and performed on 384 well plates. IC_50_ were analyzed by nonlinear regression using a four-parameter dosage-response variable slope model with the GraphPad Prism 8.4.2 software (GraphPad Software, USA). Enzyme kinetics experiments were performed using fluorescent peptide concentrations ranging from 1.25 μM to 80 μM and two different inhibitor concentrations. The activity assay was performed using 50 mM Tris-HCl pH 7.3, 20% glycerol, 1mM EDTA pH 7.3 and 0.01% triton-X. Final concentrations of 0.5 μM M^pro^ were used. The inner filter effect (IFE) was accounted for as described [43]. Data were analyzed using Dynafit [44]. Compounds structures were drawn with ACD/ChemSketch 2019.2.1 software (Advanced Chemistry Development, Canada).

## Supporting information

S1 File (PDF)
