# Supplementary material for: Biochemical screening for SARS-CoV-2 main protease inhibitors
Source: PLoS One. 2020 Oct 6;15(10):e0240079. doi: 10.1371/journal.pone.0240079 (PMC7537881; doi:10.1371/journal.pone.0240079)

Biochemical screening for SARS-CoV-2 main protease inhibitors

Camila Coelho[1*], Gloria Gallo[1*], Claudia B. Campos[1], Leon Hardy[2], Martin Würtele[1**]

[1] Department of Science and Technology, Federal University of São Paulo, São José dos Campos, Brazil. [2]Department of Physics, University of South Florida, Tampa, United States of America. *These authors contributed equally to this work. **Corresponding author.

Supporting Information

S1 Table. Kinetics of SARS-Cov-2 M^{pro} inhibitors. IC_{50}, inhibitory (dissociation) constants (K_i), Michaelis Menten constants (K_m), turnover numbers (k_{cat}), and different kinetic model candidates (together with acceptance criteria values) of SARS-Cov-2 M^{pro} inhibitors are given.

Compound	IC_{50} (μM)*	Ki (μM)*	Km (μM)*	k_{cat} (s^{-1})*	Inhibition mechanism**	SSQ§	ΔAIC&	ΔBIC#
**Thimerosal (1)**	0.6 ± 0.1	0.6 ± 0.2	47 ± 8	0.010 ± 0.0011	**Mixed**	1	0	0
					non-competitive	1.4	2.4	1.5
**Phenylmercuric acetate (2)**	0.4 ± 0.06	0.11 ± 0.03	52 ± 19	0.013 ± 0.0024	**Competitive**	1	0	0
					non-competitive	1.1	2.0	2.0
**Bronopol (3)**	4.4 ± 0.6	2.5 ± 0.3	45 ± 9	0.010 ± 0.0013	**non-competitive**	1	0	0
					mixed	1	3.0	3.9
**Tannic acid (4)**	2.1 ± 0.2	1.4 ± 0.14	47 ± 8	0.010 ± 0.0011	**Competitive**	1	0	0
					non-competitive	1.2	4.1	4.1
**Hematoporphyrin (5)**	3.9 ± 0.6	5.9 ± 0.5	50 ± 8	0.011 ± 0.0011	**non-competitive**	1	0	0
					mixed	1	3.0	4.2
					competitive	1.2	3.2	3.2
**3,4-Didesmethyl-5-deshydroxy-3'-ethoxyscleroin (6)**	10.6 ± 1.3	5.6 ± 0.5	51 ± 10	0.011 ± 0.0014	**non-competitive**	1	0	0
					competitive	1.1	2.1	2.1
					mixed	1	3.3	4.4
**Evans blue (11)**	0.2 ± 0.06	0.21 ± 0.022	56 ± 8	0.010 ± 0.0008	**Competitive**	1	0	0
**Chicago Sky Blue (12)**	7.7 ± 1.6	1.3 ± 0.2	56 ± 12	0.013 ± 0.0015	**Competitive**	1	0	0
					noncompetitive	1.2	3.2	3.2
					mixed	1	3.4	4.4

*Error values are expressed as standard error of the mean (SEM).
**Most probable mechanism according to SSQ, ΔAIC and ΔBIC analyses in bold.
§Summed squared deviation.
&Second order Akeike information criterion.
#Bayesian information criterion.

S2 Table. Toxicity of SARS-Cov-2 M^{pro} inhibitors.

Compound	LD_{50}*	Source
**Thimerosal (1)**	75 mg/kg (oral, rat)	Pesticide Chemicals Official Compendium, Association of the American Pesticide Control Officials, Inc., 1966, (1130), 1966.
**Phenylmercuric acetate (2)**	41 mg/kg (oral, rat)	Acute Toxicity Data. Journal of the American College of Toxicology, Part B., 1(175), 1992.
**Bronopol (3)**	180 mg/kg	Pesticide Index, Frear, E.H., ed., State College, PA, College Science Pub., 1969, 5(30), 1976.
**Tannic acid (4)**	No data available	
**Hematoporphyrin (5)**	307 mg/kg (IV, mouse)	Nippon Yakurigaku Zasshi. Japanese Journal of Pharmacology., 57(219), 1961.
**3,4-Didesmethyl-5-deshydroxy-3'-ethoxyscleroin (6)**	No data available	
**2,3,4-Trihydroxy-4'-ethoxybenzophenone (7)**	No data available	
**Chloranil (8)**	4 g/kg (oral, rat)	Pesticide Chemicals Official Compendium, Association of the American Pesticide Control Officials, Inc., 1966, (218), 1966.
**Plumbagin (9)**	16 mg/kg (oral, mouse)	Indian Journal of Experimental Biology, 18(876), 1980. [PMID:7461745]
**Vanitiolide (10)**	No data available	
**Evans blue (11)**	340 mg/kg (IP, mouse)	Biochemical and Biophysical Research Communications., 136(64), 1986. [PMID:3010977].
**Chicago Sky Blue (12)**	2260 mg/kg(IV, mouse)	Science, 114(41), 1951. [PMID:14854894]

* LD_{50} – median lethal dose, as reported in www.pubchem.gov

S1 Figure: **Inhibition kinetics of SARS-Cov-2 M^{pro} inhibitors.** Enzyme kinetics experiments of selected inhibitory compounds were performed with the MCA-AVLQSGFR-K(Dnp)-K-NH2 fluorescent peptide as substrate (S) and using 0.5 µM of recombinant M^{pro} and the shown inhibitor concentrations (given in µM). Fluorescence values were converted using a calibration curve with MCA to molar values and corrected for IFE (internal filter effect) to obtain enzymatic rates.

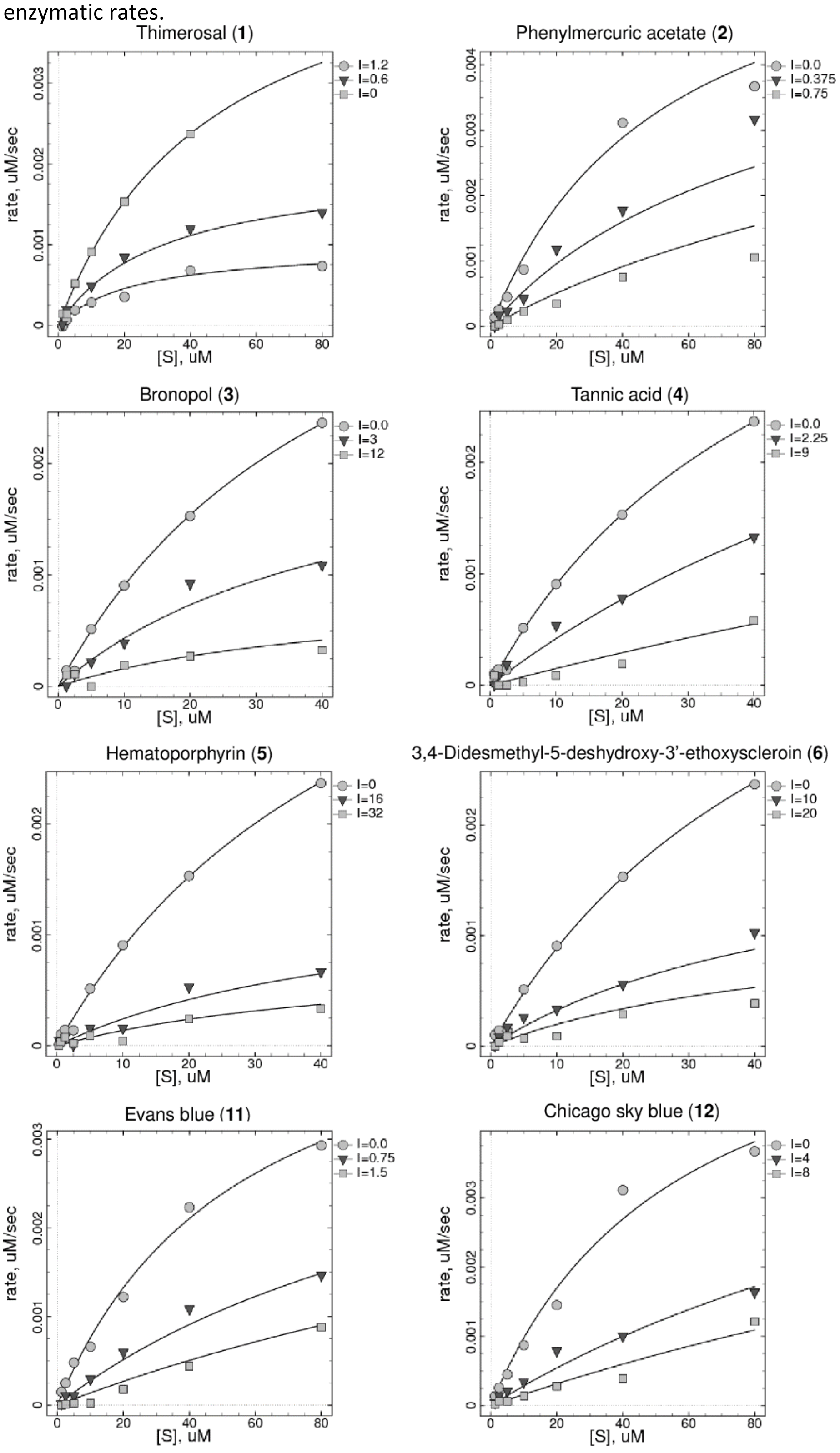

Supplement: S1 File — (PDF) [file pone.0240079.s001.pdf]
